# The acoustic and perceptual cues affecting melody segregation for listeners with a cochlear implant

**DOI:** 10.3389/fpsyg.2013.00790

**Published:** 2013-11-06

**Authors:** Jeremy Marozeau, Hamish Innes-Brown, Peter J. Blamey

**Affiliations:** ^1^Department of Medical Bionics, University of MelbourneMelbourne, VIC, Australia; ^2^Bionics InstituteMelbourne, VIC, Australia

**Keywords:** auditory streaming, cochlear implant, music training, melody segregation, hearing impairment, pitch, loudness, timbre

## Abstract

Our ability to listen selectively to single sound sources in complex auditory environments is termed “auditory stream segregation.”This ability is affected by peripheral disorders such as hearing loss, as well as plasticity in central processing such as occurs with musical training. Brain plasticity induced by musical training can enhance the ability to segregate sound, leading to improvements in a variety of auditory abilities. The melody segregation ability of 12 cochlear-implant recipients was tested using a new method to determine the perceptual distance needed to segregate a simple 4-note melody from a background of interleaved random-pitch distractor notes. In experiment 1, participants rated the difficulty of segregating the melody from distracter notes. Four physical properties of the distracter notes were changed. In experiment 2, listeners were asked to rate the dissimilarity between melody patterns whose notes differed on the four physical properties simultaneously. Multidimensional scaling analysis transformed the dissimilarity ratings into perceptual distances. Regression between physical and perceptual cues then derived the minimal perceptual distance needed to segregate the melody. The most efficient streaming cue for CI users was loudness. For the normal hearing listeners without musical backgrounds, a greater difference on the perceptual dimension correlated to the temporal envelope is needed for stream segregation in CI users. No differences in streaming efficiency were found between the perceptual dimensions linked to the F0 and the spectral envelope. Combined with our previous results in normally-hearing musicians and non-musicians, the results show that differences in training as well as differences in peripheral auditory processing (hearing impairment and the use of a hearing device) influences the way that listeners use different acoustic cues for segregating interleaved musical streams.

## Introduction

The perception and enjoyment of music engages complex brain networks (Zatorre et al., 2007) and recent evidence highlights the importance of top-down processes as well as stimulus-driven processes (Tervaniemi et al., 2009; Strait et al., 2010). Music practice has been shown to induce changes in the structure and function of the brain pathways that process sound and leads to improved performance on perceptual tasks (for example Musacchia et al., 2007). However, other uncontrolled individual differences between the musicians and non-musicians may have also contributed to the differences found in musical aptitude (see Corrigall et al., 2013 and other papers from this special edition). In this study, we sought to resolve some of these issues by removing the training difference between groups, and comparing results from groups that differ in their peripheral processing of sound. Two experiments will compare the ability to segregate auditory streams between a group of cochlear implant users and a group of normal hearing listeners.

In a typical auditory streaming experiment (Bregman, 1990), listeners are exposed to a sequence of alternating high and low notes—the sounds may be grouped together and perceived as coming from a single source (termed fusion), or perceived as streams from separate sources (termed fission). In the fusion case, the single stream is perceived as a “gallop.” In the fission case, the sequence will be perceived as two separate streams, or as a “Morse code.” The mechanism of auditory streaming and the part of the auditory pathway involved are still unclear. However, some evidence has shown peripheral cochlear (Hartmann and Johnson, 1991) and central cortical components (Carlyon, 2004). The “Peripheral Channeling” theory suggests that streaming depends primarily on the amount of overlap in the excitation pattern on the basilar membrane induced by the two stimuli; the more the two stimulus excitation patterns overlap, the more likely they are to be perceived as a single stream. A hearing impairment can affect the basilar membrane mechanisms and increases the region of excitation along the basilar membrane. Furthermore, the spread of current in a cochlear implant (CI) causes the stimulation of a wide area around each electrode. Therefore, according to the “Peripheral Channeling” theory, hearing impaired listeners should show a reduced ability to stream.

Recently many studies have highlighted the importance of the role of central processing in auditory streaming (for a review see Carlyon, 2004). This effect can be shown by studying differences caused by musical training. Marozeau et al. (2010) developed a new method to determine the perceptual distance needed to segregate a simple 4-note melody from a background of interleaved distractor notes. Results showed that for participants with musical training, the sound intensity was the most efficient cue. Compared with the spectral and temporal envelopes, only a small difference in intensity allowed a given level of melody segregation. For the non-musicians however, both intensity and spectral envelope were equally efficient streaming cues. How the acoustic cues and their perceptual correlates relate to streaming ability in CI users is currently unknown. Therefore, this study aims to examine the effects of the CI on a variety of the acoustic and perceptual cues responsible for streaming.

While CI users generally understand speech well in quiet environments, much improvement is needed for non-speech sounds such as music (for a review see McDermott, 2011), although there are some cases of CI users with good musical abilities, often associated with extensive musical training (Maarefvand et al., 2013). Unsatisfactory music perception with CIs is related to degradations in three underlying abilities: pitch discrimination, timbre discrimination, and auditory streaming ability. Unfortunately, the use of a CI degrades the acoustic cues that give rise to perceptual differences between sound sources, thus reducing the ability of the CI user to segregate different sound sources. This in turn reduces the ability to separately hear multiple lines of melody and different instruments, as well as different voices among many. In these experiments, we used a streaming task in conjunction with multidimensional scaling techniques to characterize a common perceptual distance between streams, and thus could examine streaming ability separately to the streaming difficulties caused by the degraded auditory input.

The current study is divided into two experiments. In Experiment 1, listeners were asked to continuously rate the difficulty of segregating a repeating 4-note target melody interleaved with pseudo-random distractor notes. The melody notes were always constant and repeated continuously throughout the experiment, while the distractor notes gradually changed in fundamental frequency range, intensity, temporal envelope and spectral envelope. The aim of the Experiment 1 was to determine the effect of various acoustic cues on the difficulty of segregating a melody from interleaved distractor notes. In Experiment 2, a discrete pair of melodies which differed on a combination of fundamental frequency range, intensity, temporal envelope and spectral envelope simultaneously was presented on each trial. Listeners were asked to rate the similarity between two melodies. This experiment provided a measure of the perceptual salience of each acoustic cue. While the first experiment's results are in terms of acoustic cue differences between streams, the results from the second experiment allow these differences to be re-cast in terms of perceptual differences. Taken together, the results of both experiments indicate the relationship between a perceptual difference and its ability to induce melody segregation.

## Experiment 1

Experiment 1 with CI users aimed to determine how the difficulty of segregating a repeating 4-note melody from a background of random distracter notes was affected by changes in the intensity, temporal envelope, spectral envelope, and fundamental frequency range of the background notes. This experiment is a replication of our previous study in normally-hearing listeners (Marozeau et al., 2010, 2013; Innes-Brown et al., 2011)^1^.

### Participants

A total of 12 CI recipients participated. Table 1 lists demographic and hearing-related information for all participants. All but one are native English speakers. Recruitment was conducted through the Cochlear Implant Clinic at the Royal Victorian Eye and Ear Hospital. All the participants gave written informed consent and were compensated for their travel expenses. This project conformed to The Code of Ethics of the World Medical Association (Declaration of Helsinki), and was approved by the Royal Victorian Eye and Ear Hospital Human Research Ethics Committee (Project 10-995H).

**Table 1:** **Summary of participant information**.

**ID codes**	**Rating task**	**MDS task task**	**AGE (years)**	**Sex**	**Years with implant**	**Implant**	**Etiology**	**Music training**
N1	1	1	69.9	Male	7.9	Nucleus CI24R CA	Flu—infection	No
N2	2		62.3	Female	9.3	Nucleus CI24R CA	Hearing loss from childhood, hereditary/unknown	No
N3	3	2	62.3	Male	17.3	Nucleus CI22M	Meningitis	No
N4	4		76.2	Female	9.2	Nucleus CI24M	Hereditary	No
N5	5		90.2	Female	12.2	Nucleus CI24M	Unknown—gradual hearing loss	No
N6	6	3	75.6	Male	6.6	Nucleus CI24RE CA	Gradual HL from age 50	No
N7	7		72.7	Male	6.7	Nucleus CI24RE CA	Hereditary	No
N8	8		73.1	Male	13.1	Nucleus CI24M	Meniers Disease	No
N9	9		60.8	Male	17.8	Nucleus CI24M	Hereditory/unknown	No
N10	10		71.7	Female	10.7	Nucleus CI24R CS	Unknown, gradual loss over 7 years	No
N11	11		64.2	Female	9.2	Nucleus CI24R CS	Hereditory nerve deafness	No
N12	12		81.7	Female	13.7	Nucleus 24	Hereditory hearing loss—unknown	No
N13		4	74.4	Male	11.4	Nucleus Ci24	Famillial—progressive postlingual deafness	No
N14		5	69.3	Male	4.3	freedom CA	Unknown	No
N15		6	46.5	Female	7.5	Freedom CA		No
N16		7	35.7	Female	3.7	Freedom CA	Pre-natal—unknown hereditary	No
N17		8	82	Female	4	Nucleus Ci24	Ototoxic antibiotics	No
Totals/Means	***N* = 12**	***N* = 8**	68.74 (13.1)	***F* = 9**, ***M* = 8**	9.68 (4.2)			

### Stimuli

All stimuli were 10-harmonic tones with random phase generated in Matlab 7.5 through simple additive synthesis. In Experiment 1 the procedure was controlled using MAX/MSP 5 and stimuli were played back through a soundcard (M-AUDIO ProFire 610) and loudspeaker (Genelec 8020APM) positioned on a stand at the listener's ear height, 1 m from the participant's head.

**Figure 1:**
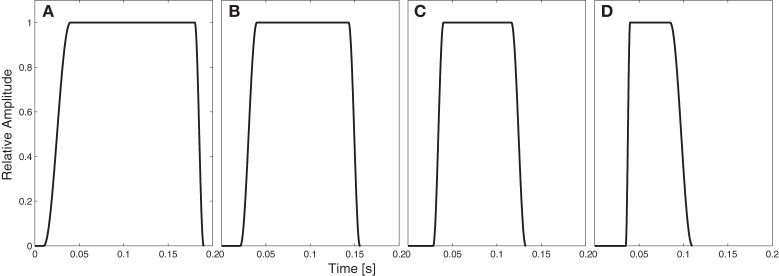
**Temporal envelope of the stimuli for different level of impulsiveness: (A) 160 ms (ratio of distractor to target impulsiveness of 100%), (B) 119 ms (ratio: 74%), (C) 90 ms (ratio: 56%), and (D) 60 ms (ratio: 37.5%)**.

**Figure 2:**
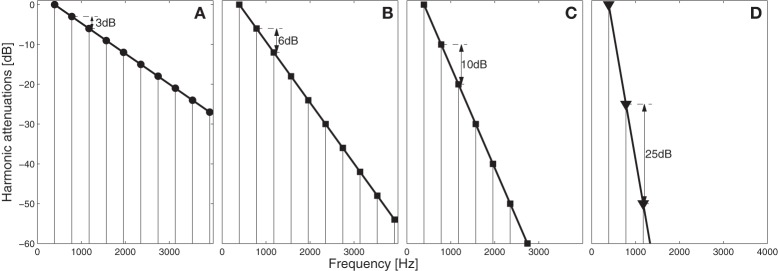
**Spectral envelope of the stimuli for different level: (A) 3 dB/harmonic (same attenuation than target melody), (B) 6 dB/harmonic (3 dB additional compared to the attenuation of target stimuli), (C) 10 dB/harmonic (7 dB additional), and (D) 25 dB/harmonic (22 dB additional)**.

The target melody was a 4-note repeating pattern with the following acoustic parameters:

1. The F0 sequence was G4-C5-A4-D5, or midinote 67–72 69 and 74. For convenience, the F0 will be represented in midinote, where each semitone is coded by consecutive integers (middle C is 60). The intervals in the sequence were chosen to be sufficiently large to allow CI recipients to discriminate between adjacent notes while also being small enough for the notes to be grouped into a single stream.
2. The temporal envelope (impulsiveness) of each note was composed of a 30-ms raised-cosine ramp on, a 140-ms sustained part, and a 10-ms raised-cosine ramp off for a total duration of 180 ms or an impulsiveness of 160 ms, defined as the full duration of the sound at half of the maximum amplitude (see Figure 1A).
3. Intensity: As impulsiveness affects loudness perception, we varied the amplitude according to a temporal loudness model (Glasberg and Moore, 2002; ANSI, 2007). Intensity was varied so that sounds with different impulsiveness were presented at the same predicted loudness. The loudness of each melody note was 65 phons (as loud as a 1-kHz tone at 65 dB SPL) in free field according to the model.
4. The spectral envelope of the notes consisted of 10 harmonics, successively attenuated by 3 dB (see Figure 2A).

### Procedure

Because auditory streaming is affected by attention and other top-down influences, the choice of task and the directions used are important. In this experiment, we were interested in the minimum perceptual difference between streams which would allow participants to segregate the two streams (for a review see Bregman, 1990). Therefore, the participants attention was directed toward segregation (“finding” the melody). It is also important to take into consideration phenomena such as the “build-up effect,” in which listeners tend to initially group signals into the same stream, with segregation slowly appearing over the first few repetitions.

The procedure used in this experiment generates a subjective measure of streaming and has been previously validated using a control experiment based on an objective detection task (Marozeau et al., 2010). During the experiment, participants continuously rated the difficulty of perceiving the target melody interleaved with pseudo-random pitch distractor notes using a slider on a midi controller (EDIROL U33, Roland Systems Group, Dee Why, NSW, Australia). The slider was labeled from 0 (no difficulty hearing the melody) to 10 (impossible to hear the melody). Participants were instructed to move the slider to the “10” position if the target melody was impossible to perceive, and to the “0” position if the target melody could be easily perceived. Ratings were re-scaled from 0–1 for analysis.

The acoustic properties of the melody notes were fixed throughout the experimental block, while a single acoustic property of the distracter notes was gradually changed. There were four conditions. In each of the conditions, either the fundamental frequency range, the intensity, the temporal envelope, or the spectral envelope of the distracter notes was gradually varied in 20 steps between a level where there was no difference between the melody and distracter notes to a level where the difference was pronounced.

1. Intensity. The intensity of each distracter note was attenuated compared to the melody notes in 20 2-phon steps from 0 to 38 phon attenuation.
2. Temporal envelope. The temporal envelope of the distracter notes was varied so that the ratio of distractor to target melody temporal envelope varied from 100 to 37.5% (see Figure 1).
3. Spectral envelope. The amplitude of each harmonic of the distracter notes was gradually decreased by the same amount in dB ranging in 20 logarithmically-spaced steps between 3 and 25 dB attenuation per harmonic (see Figure 2). In the text following the spectral envelope of the distracter notes is labeled as “additional attenuation per harmonic, ranging from 0 to 22 dB/harmonic relative to the target melody (static at 3 dB/harmonic).
4. Fundamental frequency. The 12-semitone F0 range of the possible distracter notes was gradually varied in 20 1-semitone steps from a range that totally overlapped the F0 range of the target melody to at least one octave separation between the highest distracter note and the lowest target melody note. The F0 of each distracter note was chosen randomly from this range.

The experiment consisted of blocks of trials in which the target melody was repeated continuously while a single acoustic parameter of the distracter notes was gradually varied. In order to reduce possible pitch memory effects between blocks, a pitch increment, randomly chosen for each block between 0 and 4 semitones, was added to all notes in the block. The distractor notes either started with the parameter set at the level causing the most perceptual difference from the target melody and the difference decreased (DEC blocks), or began with the target melody and distractor sharing the same physical parameters and the difference increased (INC blocks).

At the start of DEC blocks, the perceptual difference between target melody and distracter was likely to be large, and so the target and distracter were likely to be perceived in separate streams. In this case it was also likely that the target melody was easy to segregate from the distracters, with low difficulty ratings. As the parameter difference between target melody and distracter began to decrease, the distracter notes became increasingly perceptually similar to the melody, and hence the melody became more difficult to segregate. At the start of INC blocks the situation is reversed—the target melody and distractor notes shared the same acoustic parameters, and were likely to fuse into a single stream. In this case the melody would be very difficult to segregate from the distracter notes, and gradually become easier as the experiment progressed.

After every 10 presentations of the target melody (16 s), the parameter level of the distractor was either increased (INC block) or decreased (DEC block). The block ended when the parameter reached either level 19 (in INC blocks) or 0 (in DEC blocks). A paradigm where the parameter step was gradually changed was preferred over a completely randomized design in order to avoid resetting the “buildup effect” (Carlyon et al., 2001) randomly, which would occur if the parameter step were varied significantly from one trial to the next. A DEC block was always run first as a practice session, with the data from this block discarded. Following the practice session, INC and DEC blocks were run twice each, with A-B-B-A/B-A-A-B order counterbalanced across participants.

### Results

**Figure 3:**
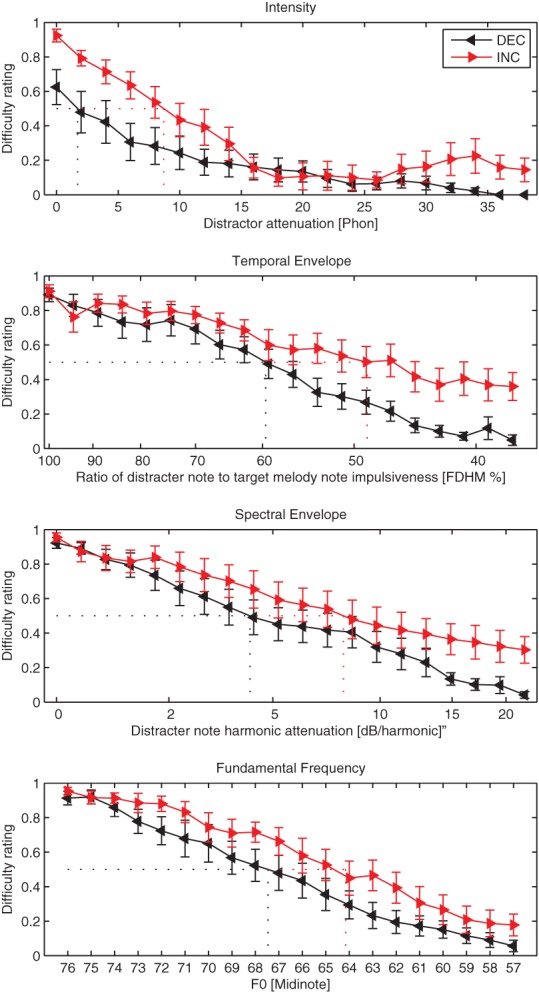
**Mean difficulty ratings as a function of the acoustic parameter in Experiment 1**.

Figure 3 shows mean difficulty ratings as a function of the parameter level in each of the four conditions averaged across the participants and repetitions. For all conditions there was a quasi-monotonic relationship between the mean difficulty ratings and the parameter level (except in the intensity INC condition for the attenuations above 25 phons). As the physical difference between the target and the distractor increased, listeners on average reported less difficulty segregating the melody from distracter notes.

In order to test the statistical significance of each acoustic parameter on the difficulty ratings, a generalized linear model (GLM) was used. All statistical analysis was conducted using R version 2.15. A GLM was performed in each condition separately using the difficulty ratings as the dependent variable. A GLM was preferred over ANOVA because it is a mixed model with categorical and continuous factors. The factor that was expected to have the most effect on the difficulty ratings was the physical difference between the target and the distractor. This factor will be called “Step” to reinforce the fact that the physical difference between the repeating melody and random distracter notes was varied with 20 intermediate steps ranging from similar (step 0) to very different (step 19). After visual inspection of the results (Figure 3) it was decided that a linear regression was appropriate to model the effect of the Step factor. Therefore, only one degree of freedom was needed. The other factors in the GLM were Context (*df* = 1, INC or DEC) and Repetition (*df* = 1, first or second repeat). The alpha level was set at.01. The results of the GLM are shown in Table 2.

**Table 2:** **Results from four GLM analyses on difficulty ratings, η^2^ (eta-squared)**.

**Analysis/Condition**	**Step (df = 1)**			**Context (*df* = 1)**			**Repetition (*df* = 1)**		
	***F*_(1, 11)_**	***p***	**η^2^**	***F*_(1, 11)_**	***p***	**η^2^**	***F*_(1, 11)_**	***p***	**η^2^**
Intensity	74.4	<0.01	0.32	15.4	<0.01	0.05	0.05	0.83	<0.01
Spectral envelope	134.4	<0.01	0.47	6.8	0.02	0.03	0.42	0.53	<0.01
Temporal envelope	61.2	<0.01	0.41	15.1	<0.01	0.05	0.06	0.81	<0.01
F0	196.9	<0.01	0.58	11.46	<0.01	0.03	0.34	0.57	<0.01

In all four analyses, neither the main effect of Repetition nor any of its interactions was ever significant. This attests to the reliability of the method as difficulty ratings did not vary from block to block. The main effect of Step was highly significant and accounted for the highest proportion of the variability of the data. The main effect of Context was highly significant in three of the conditions, however only a trend was found in the Spectral condition. This is contrary to our previous findings in normally-hearing listeners, in which the Context (INC or DEC blocks) did not affect the difficulty ratings (Marozeau et al., 2013). As hearing ability decreases it is possible that previous knowledge of the sound is more important. No interactions were found between any factors. In order to further illustrate the effect of the INC or DEC context, we used linear interpolation to estimate the magnitude of each acoustic parameter that was present when the difficulty rating was.5 (fine dashed lines in Figure 3), separately in the INC and DEC conditions. In the DEC blocks, the experiment started with a large separation between the melody and distracter notes, and so difficulty ratings generally started low and increased toward the 0.5 point, whereas in INC blocks the situation was reversed. In the Intensity condition, an average of 8.7 phons difference between the target melody and distractor notes was needed for the participants to indicate a difficulty of 0.5 in INC blocks, and only 1.7 phons in the DEC blocks. In the Temporal Envelope condition, the distractor impulsiveness at the 0.5 difficulty rating point was less than half (48.9%) of the target melody impulsiveness in the INC blocks, and 59.5% of the target melody impulsiveness in the DEC blocks. In the Spectral Envelope condition, each successive harmonic was attenuated by an additional 8 dB/harmonic over the target melody in the INC blocks and an additional 4.3 dB/harmonic in the DEC blocks. In the F0 condition, the distractor ranged between midinote 64–56 in the INC blocks and between 67 and 58 in the DEC blocks. Overall, at the 0.5 difficulty rating point the distracter and target melody notes were much closer in each acoustic parameter when the task started easily and was gradually getting more difficult than vice versa.

### Discussion

#### The effect of intensity

The results in the intensity condition showed that the attenuation of the distracter notes had a large and significant effect on the difficulty ratings, and that the pattern of this effect was different according to the context (between the INC and DEC blocks). In the DEC blocks (see Figure 3 top panel), the experiment started with the maximum distracter attenuation (38 phon—black line, starting on the far right of the x-axis). At this point the distracter notes were of low intensity (27 phon) and the difficulty ratings for segregating the melody were very low. As the experiment progressed (moving leftwards on the x-axis), the distracter notes became louder, and the difficulty ratings began to increase up to a final average difficulty of ~0.61 when the melody and distracter notes were presented at equal intensities. In the INC blocks however (red line, with the experiment moving from left to right on the figure), the pattern of difficulty ratings was not simply the reverse. At the beginning of the INC blocks, even though the stimuli were the same as at the *end* of the DEC blocks, the average difficulty rating was almost the maximum (compared to.61 for the end of the DEC blocks).

In order to understand the relationship in CI users between the physical property of intensity in phons, and perceptual loudness in sones, it is useful to understand how sound pressure level is mapped to stimulation current level and a predicted loudness in a CI. All the CI brands work slightly differently, but as all participants in this study were fitted with the same brand (Cochlear Ltd.), only this implant will be addressed. To set the relationship between sound pressure level and current level, the clinician first increases the current level of a single channel until the patient starts to perceive a sensation. This threshold level (T-level) is usually associated with sounds at 25 dB SPL. Then the audiologist increases the current level until the patient reports a comfortably-loud level (C-level) which is associated with a sound at 65 dB SPL. Therefore, any sounds below 25 dB SPL will not be transmitted, and sounds above 65 dB SPL will be limited to the C-level. At distracter attenuation levels of ~20–25 phon attenuation, the difficulty ratings reached a minimum. In the pitch range of the experiment, this level of attenuation resulted in distracter notes that were below 25 dB SPL, too low for the CI users to perceive.

These results highlight the importance of the previous context in the melody segregation task—when the listeners had a chance to hear the melody with distracter notes at a very low intensity at the beginning of the experiment, they were able to hear the melody more easily as the experiment progressed and the distracter notes became louder, even in the most challenging conditions. If they were presented with the most challenging conditions first, however, the task was more difficult overall (not only at the start). In addition to the overall higher difficulty ratings in INC compared to DEC blocks, the average difficulty ratings in INC blocks did not follow a strictly monotonic pattern—they gradually decreased as the distracter attenuation increased, but then surprisingly started to increase again once the attenuation increased over 25 phons. Neither of these results were found in our earlier study in normally-hearing listeners (Marozeau et al., 2013).

In the INC blocks, the increase in difficulty ratings at distracter attenuation levels above 25 phons is still surprising. However, an inspection of the individual responses revealed that only 4 of the 12 participants showed this non-monotonic pattern (2 showed in only one repetition, 1 had consistently high difficulty ratings at all attenuation levels, and only one showed this behavior on both repetitions). It is possible that some participants might have been confused by the fact that they did not hear any distractors toward the end of the INC blocks. The analysis was repeated but only including attenuation steps larger than 29 phons and no effect of step was found. Therefore, the change in difficulty at these levels is not related to the change in attenuation of the distractor.

Another surprising result was the fact that the distracter attenuation level at the.5 difficulty rating point was less than that found in our previous study with normally-hearing listeners (Marozeau et al., 2013). For the CI users in this study, the distractor needed to be attenuated by 8.7 phons to reach the.5 difficulty rating point in the INC blocks and only by 1.7 in the DEC blocks, compared to an average of 10.2 phons for the normally-hearing listeners (Marozeau et al., 2013). The normally-hearing listeners required more attenuation of the distracter notes in order to reach the 0.5 difficulty rating point than the CI users. This apparently paradoxical result can be explained in terms of the loudness growth function applied by the CI sound processor. Based on McKay et al. (2003) the loudness growth function is steeper in CI users than in normally-hearing listeners at very high levels (approximately halving the loudness every 5 dB) and gradually becomes shallower and close to the slope for normal hearing at mid-levels (halving the loudness every 10 dB). Therefore, the lower difficulty ratings for CI users might not suggest that they require less loudness difference to segregate the melody, but rather reflects the fact that due to the steeper loudness growth function with electric stimuli they experience more change in loudness for a given physical change than in normal hearing.

#### The effect of the temporal envelope

Studies on the music abilities of CI listeners often report poor perception of pitch but preserved perception of rhythm (for a complete review see McDermott, 2004). This can be explained partly by the sound-processing employed in CIs. The input sound is filtered into 22 channels with center frequencies between 125 and 8000 Hz. In each channel the amplitude of the temporal envelope is used to derive the current level of biphasic pulses delivered at a rate varying from 250 to 3500 pulses per second (pps, typically set to 900 pps). At 900 pps, a pulse will be sent every 1.1 ms.

This period is less than the measure of temporal resolution in normal hearing listeners measured by the smallest detectable gap length ranging from 2 to 20 ms (for a review see Verhey, 2010). The durations of the stimuli used in the current study varied from 60 to 160 ms FDHM. Therefore, the sound processor itself does not impose any limitations on the perception of the different temporal envelopes used in the current study.

Overall the results in the INC block of the CI participants are very close to our previous results in normally-hearing non-musicians, who needed a distractor to melody impulsiveness ratio of 52% to reach the.5 difficulty rating level. In the DEC blocks the results are closer to the musicians who needed a distracter to melody impulsiveness ratio of 62%.

#### The effect of the spectral envelope

Compared to normal hearing listeners, CI listeners needed a larger difference in spectral envelope to perceive the target melody. This result shows the difficulty that CI listeners have in perceiving timbre difference based only on spectral cues.

The target stimuli were composed of 10 harmonics with 3 dB attenuation per harmonic. The F0 was presented at between 47 and 53 dB SPL. Therefore, the tenth harmonic was presented at levels from 20 to 26 dB SPL and was unlikely to be processed, as most T-levels were set at 25 dB SPL. The number of harmonics processed was highly dependent on the additional attenuation. With 1 dB additional attenuation, only 6 out 10 harmonics will be presented at levels above 25 dB SPL. With 3 dB additional attenuation only 4 harmonics will be processed. And after 8 dB only two harmonics will be processed. It should then be theoretically possible to discriminate the target and the distractor by focusing on that last harmonic.

#### The effect of fundamental frequency

The results of the fundamental frequency condition show that the 0.5 difficulty rating points were reached when the distractor ranged between midinote 56 and 64 in the INC blocks and between 58 and 67 in the DEC blocks. As the target melody ranged between 67 and 74, three semi-tones separation was needed in the INC blocks to allow the melody to be segregated from the distracter notes. This difference corresponds to the minimum F0 separation needed for CI listeners to be able to discriminate two notes (Gfeller et al., 2002). In the DEC blocks, the 0.5 difficulty rating was reached with one note overlap between the target and the distractor. This result is similar to normally-hearing non-musician listeners (Marozeau et al., 2010), suggesting that despite degraded hearing, CI users report similar difficulty ratings in this task when the context is clearly outlined.

#### The effect of context

The context factor affected difficulty ratings across all the acoustic cues. The change in difficulty ratings as each acoustic parameter changed depended on the direction of the change in the acoustic cues. This was significant for intensity, temporal envelope and F0, but not for the spectral envelope.

That the context had a large and significant effect was unexpected. It might be speculated that as in their everyday life experience, hearing impaired listeners compensate for their hearing loss by using any possible cues, such as visual information, syntactic context, or the distracter note context information available in this experiment.

The effect of age should also be considered. The CI and NH listeners differ in their average age (CI mean age 71.7 years, SD 8.7 years, NH mean age 28.0 years, SD 4.8 years). It might be possible that both groups differ in their auditory working memory ability. As the target melody was required to be learnt, the younger NH group could remember it more easily and did not need extended repetitions with no distractors as with older CI groups in the DEC condition.

## Experiment 2

Experiment 1 reported the amount of physical change in four acoustic cues that was required for CI users to be able to segregate a repeating melody with a difficulty rating of 0.5. Experiment 2 aimed to recast these physical differences in terms of perceptual salience using a dissimilarity rating paradigm and multi-dimensional scaling analysis (MDS). We have previously used these techniques in normally-hearing listeners (Marozeau et al., 2013).

### Participants

A total of 8 CI recipients participated in Experiment 2 (including 3 who participated in Experiment 1). Table 1 lists demographic and hearing-related information for all participants. The recruitment was conducted through the Cochlear Implant Clinic at the Royal Victorian Eye and Ear Hospital. All the participants gave written informed consent and were compensated for their travel expenses.

### Stimuli and procedure

In Experiment 2, the target melody from Experiment 1 was presented twice in succession, with no distracter notes. The first melody in the pair differed from the second by 1, 2, 3, or 4 of the same acoustic parameters as in Experiment 1 simultaneously (fundamental frequency, intensity, and the spectral and temporal envelopes). Sixteen of these combinations were randomly chosen and constructed. The stimuli were presented at an intensity step of either 65, 63, 61, 59, 57, or 55 phons, with an additional attenuation per harmonic of either 3.55, 2.86, 2.24, 1.69, 1.19, 0.75, 0.35, or 0 dB, a FHDM of either 119, 126, 133, 142, 150, or 160 ms and a base F0 (of the first note) ranging from C3 to G3.

Experiment 2 consisted of a training phase and an experimental phase. In the training phase, listeners were presented with each of the 16 stimuli in random order to acquaint them with the range of possible differences in the set of stimuli. The listeners controlled the delivery of the stimuli by pressing one of 16 buttons (one for each stimulus), and were instructed to continue listening as long as they wanted to after each stimulus was heard at least once.

In the experimental phase, the participants were presented with the same stimuli composed of every possible pair of the 16 stimuli in random order, totaling 120 pairs. A single pair was presented in each trial. In each trial, the participants were instructed to judge how similar the pairs were, and to respond by moving a cursor on a slider bar labeled from “most similar” to “least similar.” Listeners could listen to the pair as many times as they wanted, by pressing a “listen again” button. When they were satisfied with their judgment, they pressed a “validate” button, and the next trial began. The order within pairs and the order of pairs was random, and a different randomization was used for each session and subject. For each pair presented, the dissimilarity response was stored in a matrix as a continuous value ranging from 0 (similar) to 1 (different).

As the factor step is a different physical quality between conditions, it is not possible to compare the slope of each condition. One needs to be able to convert the physical scale to perceptual distance, which can using the MDS method detailed below.

The experimental interface and the data collecting software were implemented in MAX/MSP 6. Stimuli were played back through a soundcard (MOTU I/O-24) and loudspeaker (Genelec 8020APM) positioned on a stand at the listener's ear height, 1 m from the participant's head.

### Analysis

The dissimilarity matrix of each participant was first analyzed through a cluster analysis to identify any possible outliers. No outliers were identified so all the matrices were averaged together. Because the stimuli were designed based on four clear physical dimensions known to induce four corresponding perceptual dimensions, the MDS algorithm was constrained to match an expected physical space created based on the characteristic of the stimuli using the SMACOF package in R.2.15.3. This constraining differs from classical MDS methods where the distance between each pair of stimuli is derived by all the dissimilarities measured within the set of the stimuli.

By constraining the MDS, the correlation between the perceptual and physical dimension was maximized (i.e., the slope between the physical parameter and its perceptual association). Although the order of each stimulus was constrained on each dimension, the relative contribution of each dimension (the slope of each dimension) was not.

As in a classical MDS, the physical solution was derived by minimizing the stress function *S*:

(1) $$S={\sum_{i<j\leq n}\left({\text{d}}_{ij}-\partial_{ij}\right)}^{2}$$

where ∂_*ij*_ is the averaged dissimilarity judged between the stimuli *i* and *j* and *d*_*ij*_ is the physical distance extracted from the 16 ∗ 4 output matrix *Y*:

(2) $$d_{ij}=\sqrt{\sum_{n=1}^{4}}{\left(y_{in}-y_{jn}\right)}^{2}$$

where *y*_*in*_ is the element [*i,n*] of *Y*.

The output configuration *Y* is constrained to be the results from the multiplication of a 16 × 4 matrix *X* by a diagonal 4 × 4 matrix, *C*.

(3) Y = XC

Where each of the 4 columns of *X* corresponds to a physical dimension and each row represents the physical characteristic of the stimulus on those dimensions.

### Results

**Figure 4:**
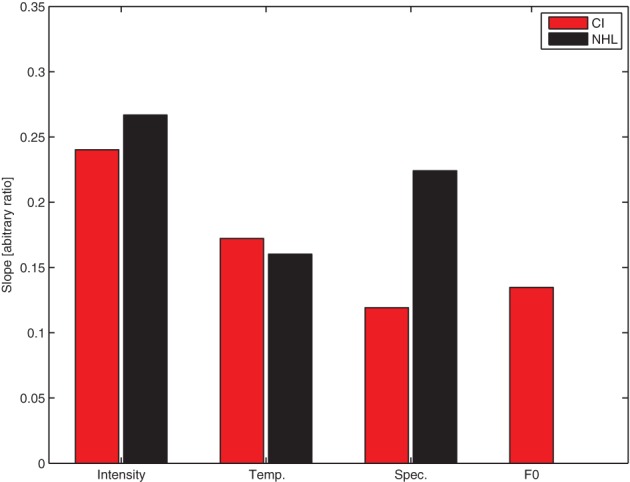
**The slope of the regression line between the physical parameter step and the MDS coordinate for the cochlear implant users in each condition in the current study (red bars), compared with the same results from our previous study in normal hearing listeners (Marozeau et al., 2013, black bars).** The F0 was not tested in the normal hearing listeners (NHL).

Figure 4 shows the slope of the regression line between the physical parameter step and the MDS dimension for the CI users in each condition in the current study, compared with the same results from our previous study in normal hearing listeners (Marozeau et al., 2013, note that the slope for NH listeners has been re-calculated for this graph using the constrained MDS technique used in the current study).

The slope indicates how much physical change is needed in order to reach a defined perceptual distance. Note that a difference of a given number of physical steps does not necessarily correspond to the same magnitude of perceptual difference for different acoustic cues, so it is not meaningful to compare the slope between conditions, but it is possible to compare the slope between groups. Of particular note, the slope in the Spectral Envelope condition for the NH listeners is about twice the slope for the CI users, meaning that twice the number of physical steps in the Spectral Envelope condition were required to reach the same perceptual magnitude change for CI users compared to NH listeners.

**Figure 5:**
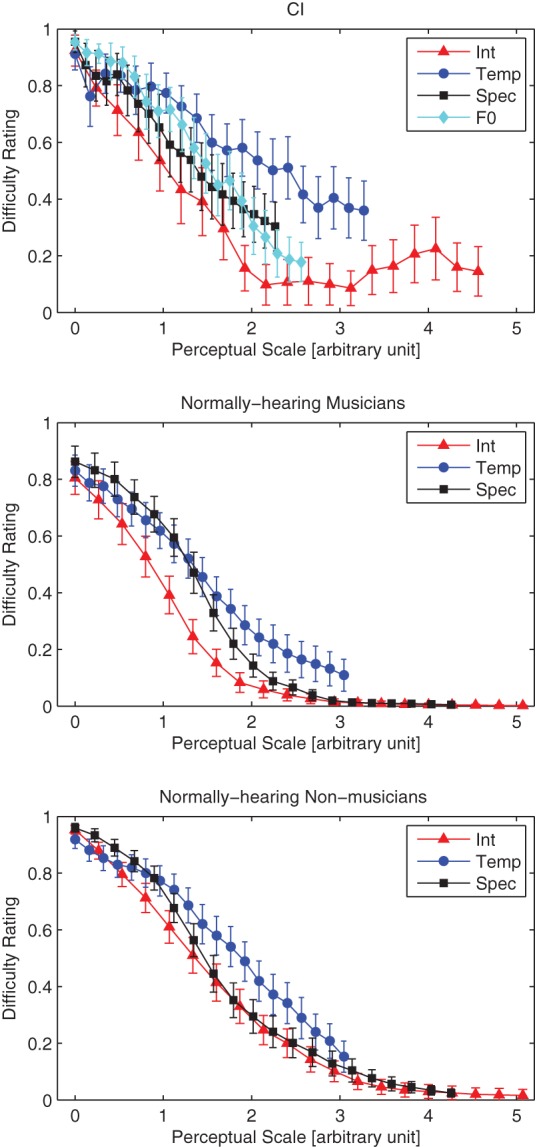
**Replots the difficulty ratings from Experiment 1, but with a different abscissa based on a perceptual scale determined from dissimilarity ratings (Experiment 2).** The error bars show the standard error of the mean.

Based on the slopes of the functions relating physical parameter changes with perceptual difference ratings found through the MDS analysis in Experiment 2, the results of Experiment 1 were re-scaled into perceptual distance units on the x-axis (Figure 5). The top panel of Figure 5 shows the results from CI users in the four conditions of Experiment 1 on the same perceptual scale. As the context had a significant effect, only data from the INC blocks is shown. In these blocks the experiment starts with the target melody and distracter notes sharing the same physical parameters, so all conditions start with the target melody very difficult to perceive.

Figure 5 shows that for CI users (top panel), intensity is the most efficient stream segregation cue i.e., the smallest perceptual difference in intensity reduces the difficulty of segregating the melody from the distracter notes. The opposite is true for the temporal envelope—a perceptually-large change in impulsiveness is needed to allow the melody to be segregated from the distracter notes.

The analysis from Experiment 1 was repeated, except that the dependent variable is now the perceptual distance and not the acoustic cue step level. In order to avoid missing data in some conditions only perceptual distances up to a value of 2.3 were considered. The analysis revealed that (1) difficulty ratings in the Intensity condition were lower than the three other conditions (*p* < 0.001), (2) difficulty ratings in the Temporal Envelope condition were larger than the three other conditions (*p* < 0.001), and (3) difficulty ratings in the F0 condition were not significantly different from the Spectral Envelope condition (*p* = 0.14).

### Discussion

Figure 4 shows that in the Intensity condition, the same amount of physical change induces a larger amount of perceptual change in CI users compared to NH listeners. This increase is in agreement with the literature suggesting that at comfortable levels CI users experience a steeper loudness growth function (McKay et al., 2003). In the Temporal Envelope condition the slope for CI users and NH listeners is similar. This confirms previous suggestions that temporal envelope cues are transmitted well by CIs, and also suggests that a given change in temporal envelope cue is perceived similarly for CI users and NH listeners. Finally the slope of the spectral envelope for CI users is about half the slope of the NH listeners. In order to induce the same change on the perceptual dimension of brightness (correlated with spectral centroid), the physical difference needs to be double in CI listeners compared to NH listeners.

## Overall discussion

Figure 5 shows the data from NH musicians and non-musicians from our previous study, re-analyzed to follow the analysis in the current study (lower panels). As with the CI listeners in the current study, the temporal envelope cues required more perceptual difference between melody and distracter for the listeners to be able to segregate the melody from distractor notes. We previously speculated (Marozeau et al., 2013) that temporal envelope cues required the most perceptual difference in order to induce streaming because of the great variability between the durations of phonemes in spoken language. Durations between adjacent phonemes can vary greatly without causing the single speech stream to segregate into two. The temporal variability of English speech was measured by Patel and Daniele (2003) by using a measure of “normalized Pairwise Variability Index” *nPVI*:

(4) $$nPVI=\frac{100}{m-1}\times \sum_{k=1}^{m-1}\left|\frac{2\left(d_{k}-d_{k+1}\right)}{\left(d_{k}+d_{k+1}\right)}\right|$$

where *m* is the number of vocalic intervals (sequences of consecutive vowels that might or might not belong to the same syllable or word) or musical notes in an utterance, and *d*_*k*_ is the duration of the *k*th interval. It is possible to convert the impulsiveness ratio, *r* into an equivalent *nPVI* using the following equation:

(5) $$nPVI=2\frac{1-r}{1+r}$$

In the DEC blocks, the nPVI required for CI users to judge that the melody was easy to hear (rating at 0.5 difficulty) was 66.66 which is similar to the nPVI found for English language (66.99). In musicians with normal hearing the nPVI was 47.3. This result is very similar to the nPVI of English classical music, 46.91(Patel and Daniele, 2003). Normally-hearing non-musicians also show a nPVI of 63.7, closer to the nPVI of English speech. As CIs accurately transmit the temporal envelope of sounds, these observations suggest that CI recipients, similarly to non-musician NH listeners, start to segregate two streams when the difference between them is larger than the average temporal variability characteristics of speech.

It has also been shown that the statistical regularities of sound signals can be used by the brain to derive the probability of the next item in a sequence of auditory events (for a review see François and Schön, 2013), and may serve as the basis of language or music learning. In normal hearing, the learning of these regularities can occur through passive exposure, or actively via music training. Similarly, CI users must re-learn the new statistical properties of the auditory sensation delivered via their implant.

## Conclusions

The most efficient streaming cue for CI users was loudness (the perceptual correlate of intensity). This was also the case in our previous study in normal hearing listeners with musical background. On the other hand, similarly for the normal hearing listeners without musical background, a greater difference on the perceptual dimension correlated to the temporal envelope is needed for stream segregation in CI users. No differences in streaming efficiency were found between the perceptual dimensions linked to the F0 and the spectral envelope.

Combined with our previous results in normally-hearing musicians and non-musicians, the results show that differences in training as well as differences in peripheral auditory processing (hearing impairment and the use of a hearing device) influences the way that listeners use different acoustic cues for segregating interleaved musical streams. We have previously shown that musical practice reduces the difficulty of segregating a melody from distracter notes.

In this study we assessed stream segregation ability, but analyzed the stimuli in terms of the *perceptual difference* between streams, thus taking into account the difference in peripheral hearing components and processes between CI and NH listeners. Although there were differences in streaming ability between the groups, CI users were able to use perceptual differences generated by each different acoustic cue in order to segregate the melody. This may indicate that plasticity of central processes related to stream segregation compensates for peripheral loss.

### Conflict of interest statement

The authors declare that the research was conducted in the absence of any commercial or financial relationships that could be construed as a potential conflict of interest.

## Footnotes

^1^A portion of the data in the fundamental frequency condition has already been published (Innes-Brown et al., 2011). However, the data were analyzed differently in this paper.
